# Supplementary figures and images for: Deciphering Network Community Structure by Surprise
Source: PLoS One. 2011 Sep 1;6(9):e24195. doi: 10.1371/journal.pone.0024195 (PMC3164713; doi:10.1371/journal.pone.0024195)

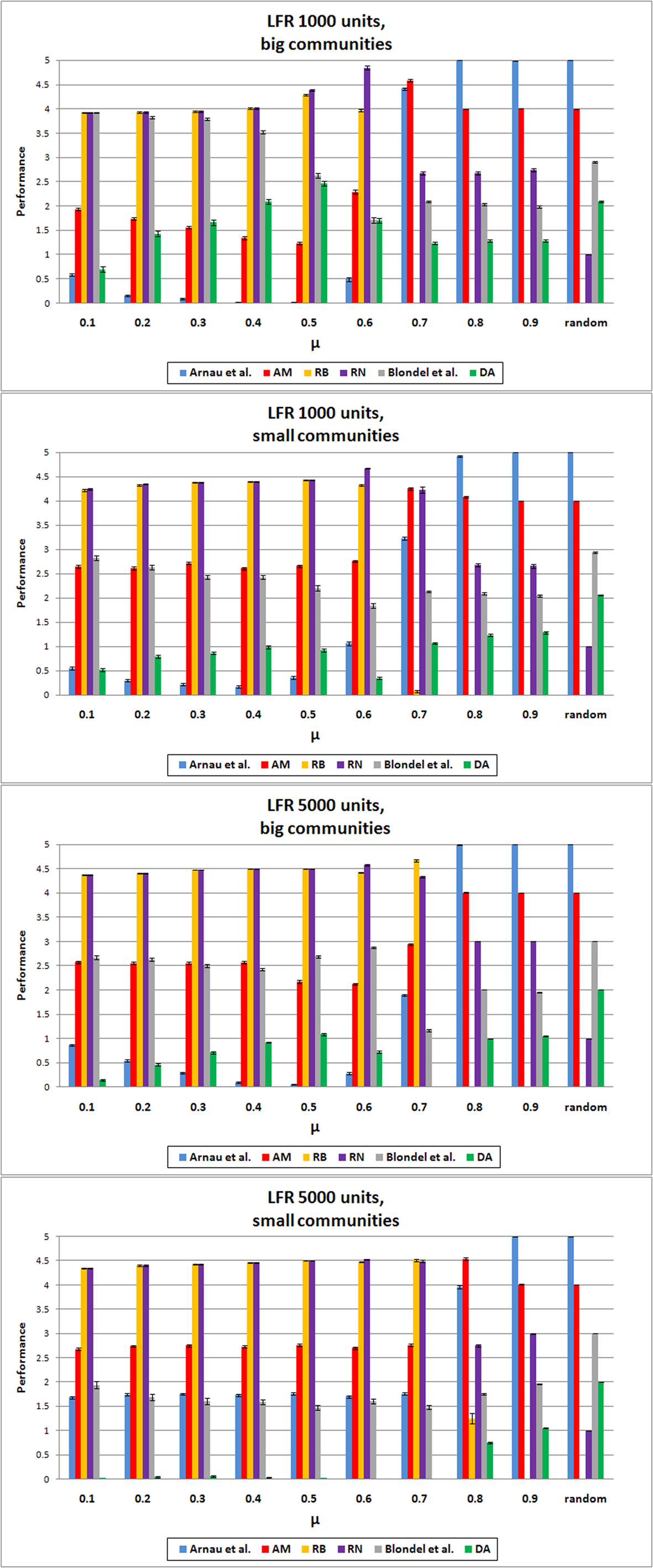

Supplement: Figure S1 — Average performances of the algorithms in the LFR benchmarks. With different network sizes (1000, 5000 units), community sizes (small: 10 to 50 units per community; big: 20–100 units per community) and values of mixing parameter (μ) and for random networks of the same size. After ordering the algorithms from best to worst performance, their ranges were added for the 100 different networks. Performance is defined as P = 6 - average range. (TIF) [file pone.0024195.s001.tif]

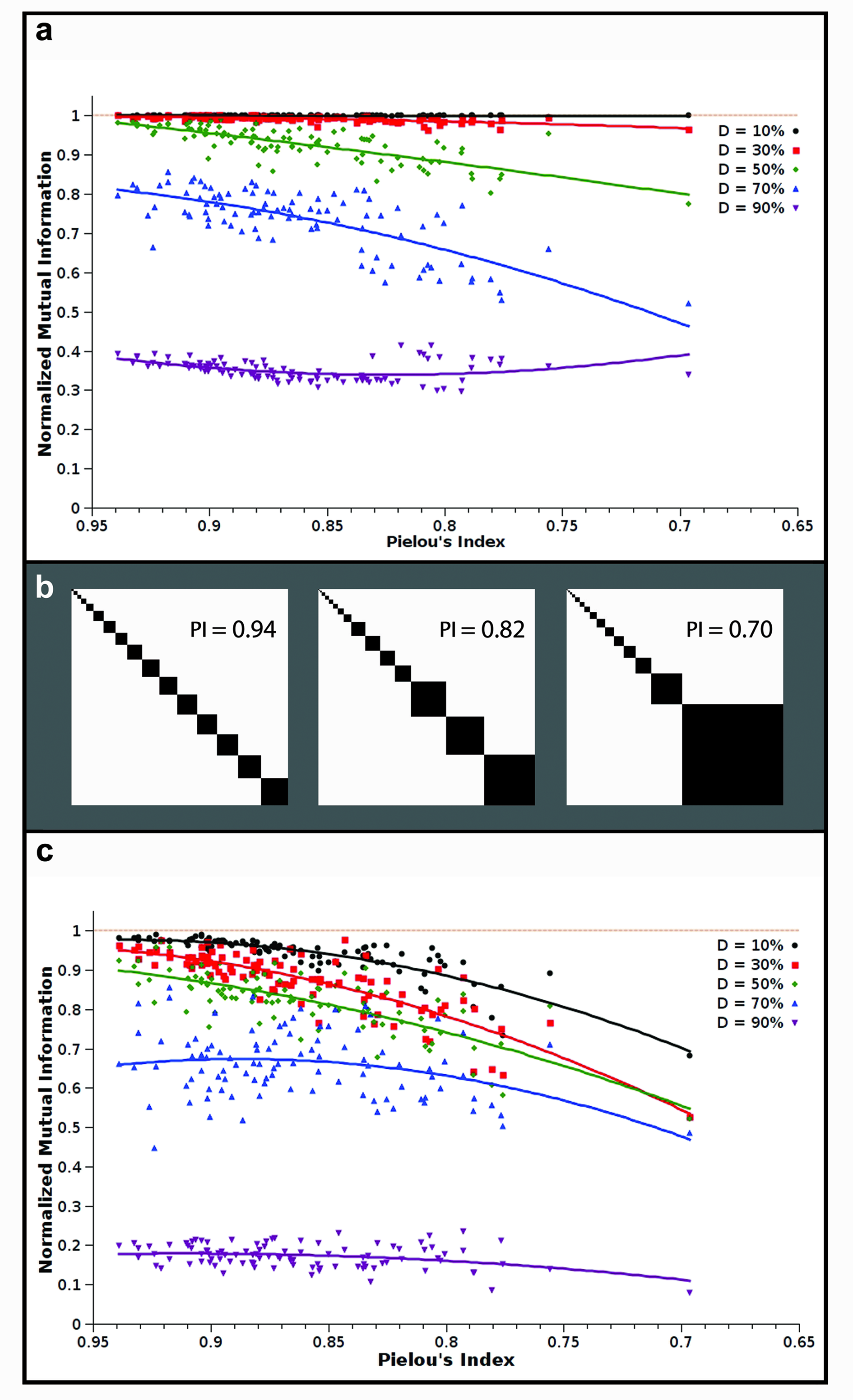

Supplement: Figure S2 — Details of the results for the RC benchmark. a) Normalized Mutual Information values for the 100 networks tested, obtained by S maximization. Given that both a low Pielou's index and high D may alter the original structure of the network, these results would tend to underestimate the real quality of the partition into communities obtained. Lines correspond to the second degree polynomials that best fit the results, which were found to be better than the first degree ones. b) Examples of the relative sizes of communities for different Pielou's indexes, to show the very different structures provided by generating the community sizes according to a broken stick model. c) Summary of the results in the RC benchmark with Q maximization. The results are much worse than those shown in panel a), due to the resolution limit that affects Q values when some communities are small (low Pielou's indexes). Lines again correspond to the best fits according to second degree polynomials. (TIF) [file pone.0024195.s002.tif]

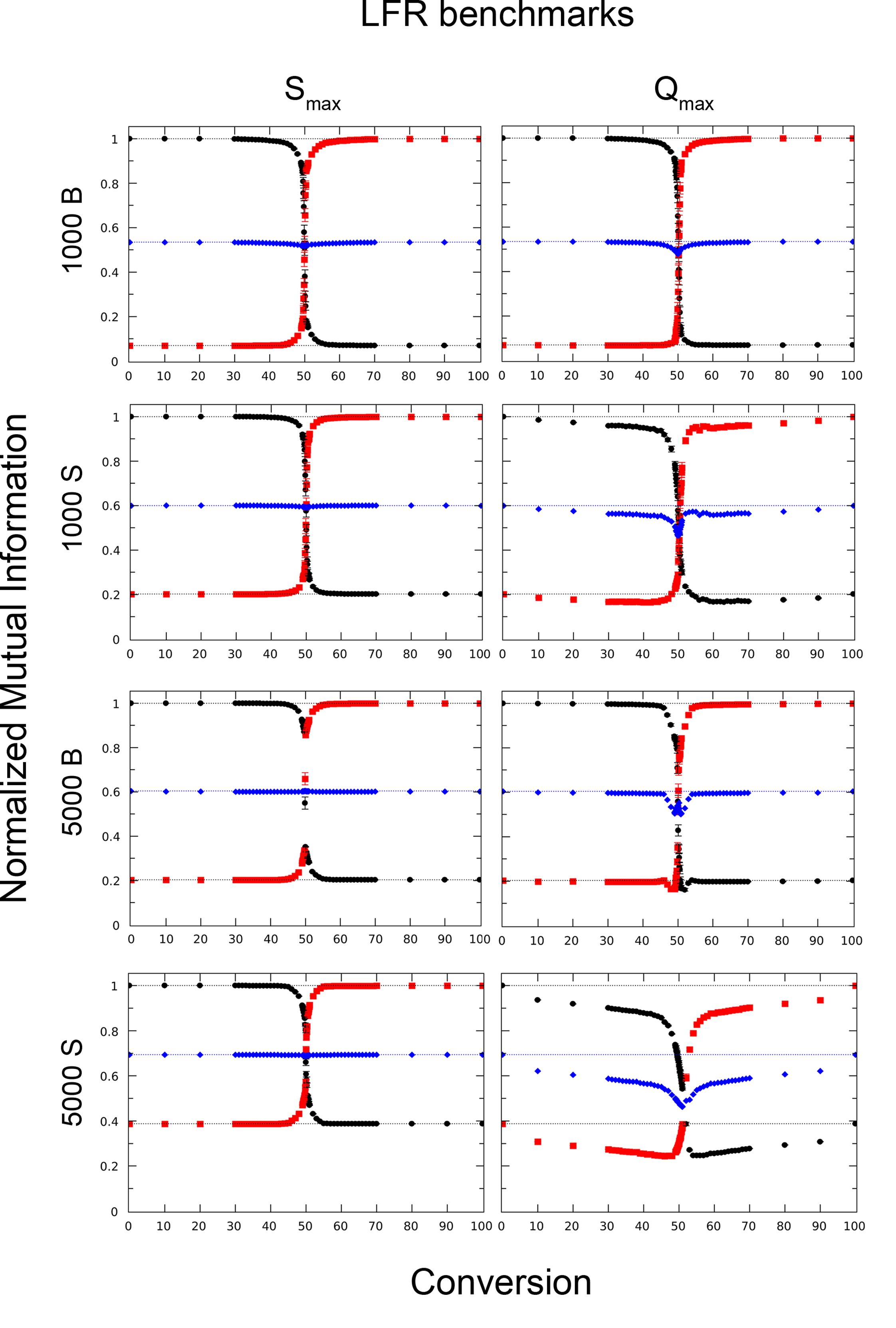

Supplement: Figure S3 — Behavior of S and Q maximization in closed LFR benchmarks. Notice the obvious decrease below (1+NMIIF)/2 when Q is maximized. (TIF) [file pone.0024195.s003.tif]

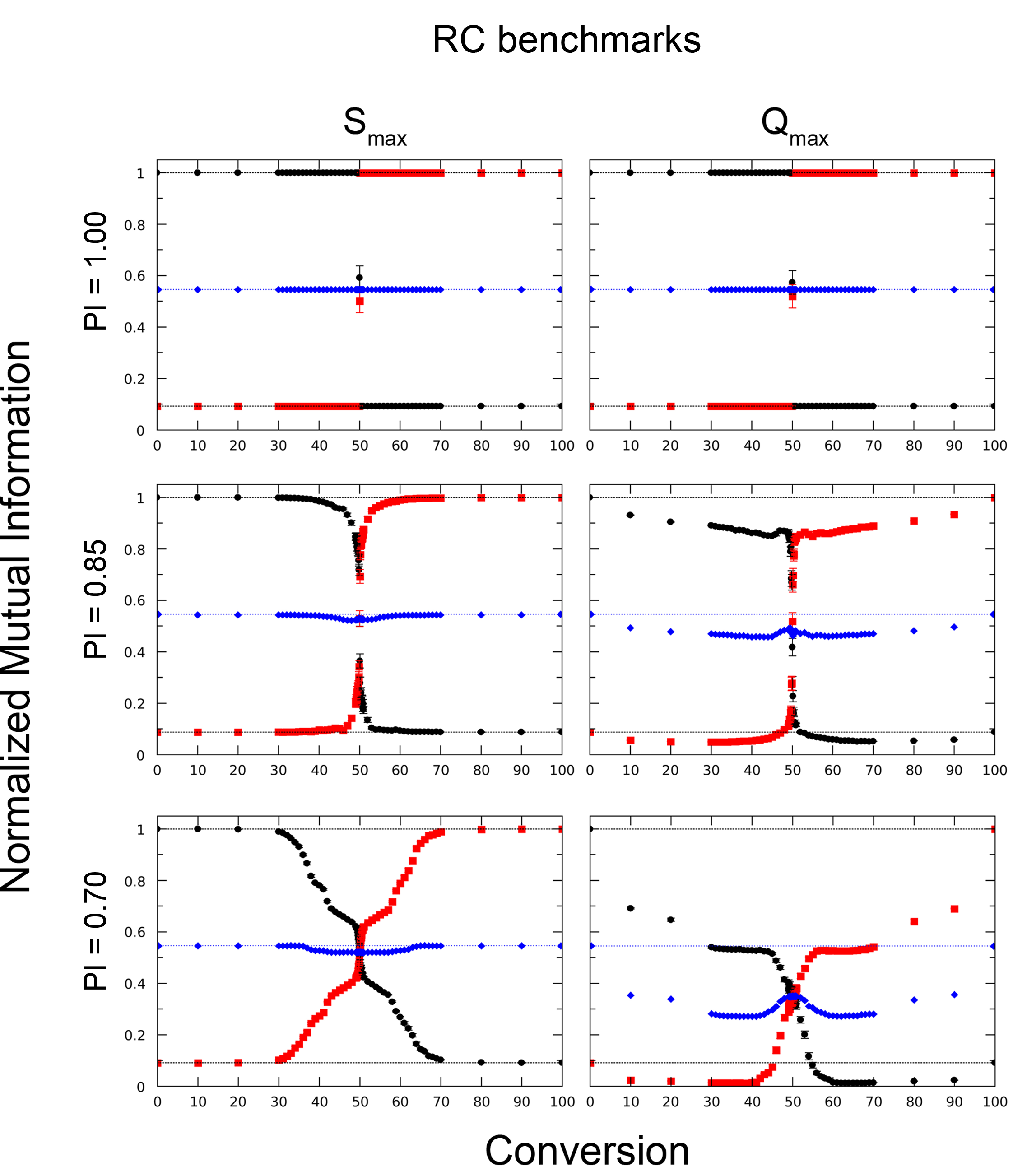

Supplement: Figure S4 — Results for S and Q maximization in the closed RC benchmarks. The behavior of Smax is again qualitatively better than the one of Qmax, except when all communities are identical. (TIF) [file pone.0024195.s004.tif]

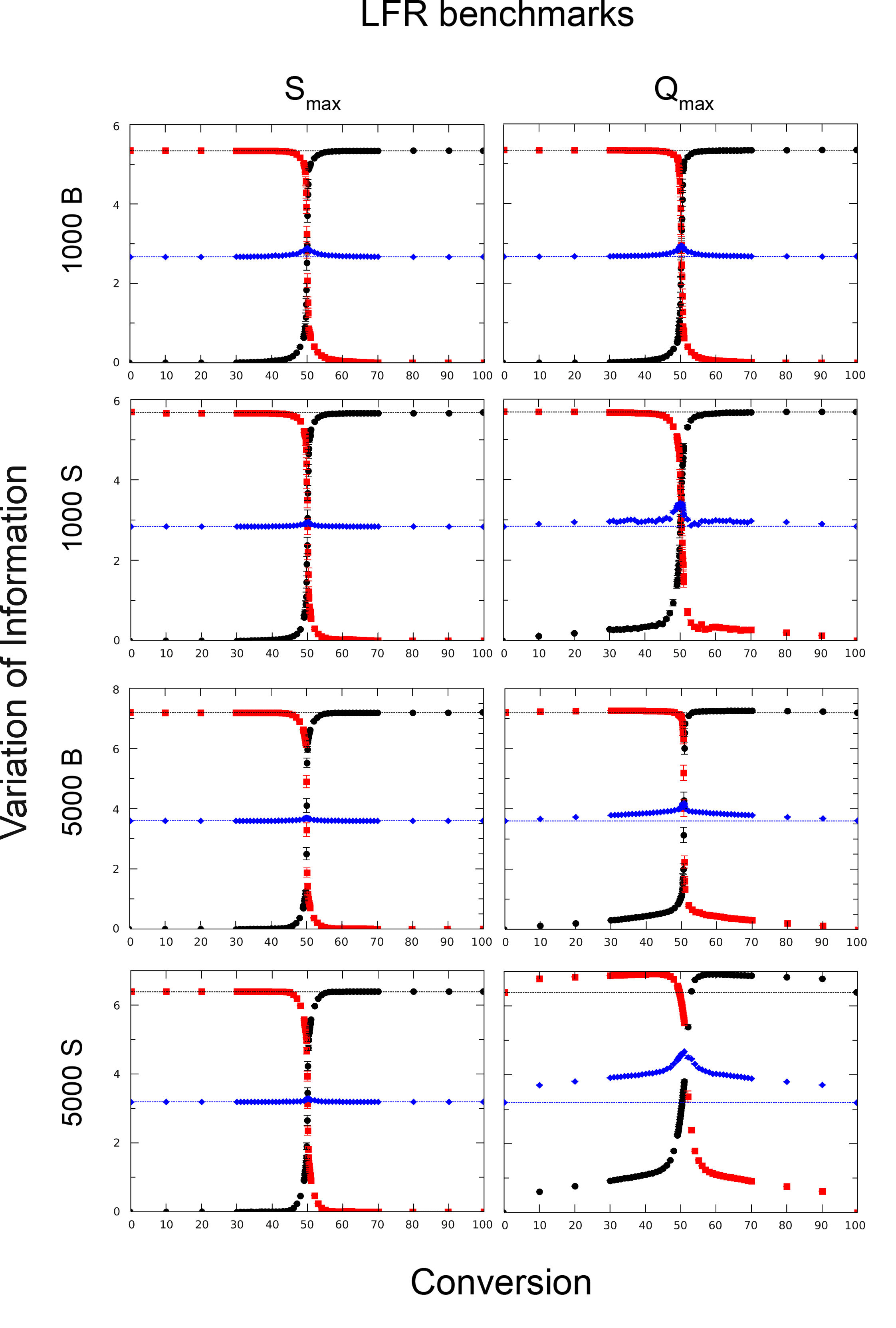

Supplement: Figure S5 — Behavior of S and Q maximization in closed LFR benchmarks using Variation of Information (VI) as a measure of congruence. As it can be deduced from Formula [4] in the main text, a good behavior of a parameter implies minimal deviations from the expected value VIIF/2 (blue line). Results are almost identical to those shown in Figure S3 using NMI. S max behavior is clearly better than Qmax behavior. (TIF) [file pone.0024195.s005.tif]

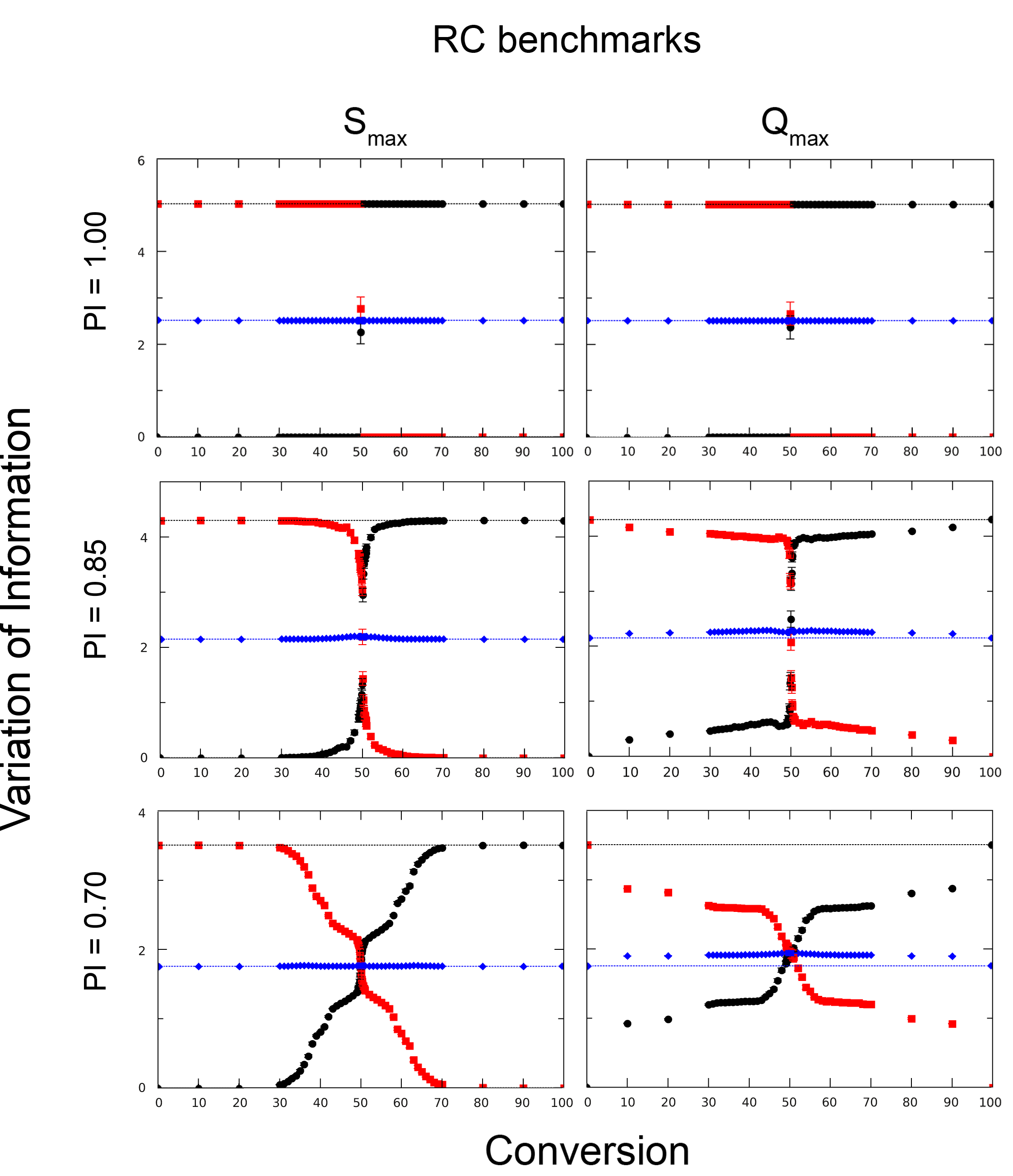

Supplement: Figure S6 — Results for S and Q maximization in the closed RC benchmarks, measured with VI. The behavior of S max is again qualitatively better than the one of Qmax, confirming the results shown in Figure S5. (TIF) [file pone.0024195.s006.tif]
