# Supplementary material for: Deciphering Network Community Structure by Surprise
Source: PLoS One. 2011 Sep 1;6(9):e24195. doi: 10.1371/journal.pone.0024195 (PMC3164713; doi:10.1371/journal.pone.0024195)
Supplement: Table S1 — Detailed results obtained for the LFR benchmarks. The values of NMI when S and Q are maximized are indicated, together with the percentage of cases in which NMI = 1 and the values of S max and S orig (i. e. the S value obtained assuming that the original structure is present). Notice that when μ = 0-6-0.7, S max>Sorig, meaning that the original structure is not the one present anymore. In those cases, NMIs are expected to rapidly decrease, as indeed is observed. (DOC) [file pone.0024195.s007.doc]

**Table S1**

| **LFR Benchmark** | **μ** | **NMI ± s. e. m.** | | **NMI_S_ = 1 (%)** | ***S*_max_ ± s. e. m.** | ***S*_orig_  ± s. e. m.** |
| --- | --- | --- | --- | --- | --- | --- |
|  |  | ***S*** | **Q** |  |  |  |
| **1000 units, big communities** | **0.1** | 1.000 ± 0.000 | 1.000 ± 0.000 | 100 | 9918 ± 52 | 9918 ± 52 |
|  | **0.2** | 1.000 ± 0.000 | 1.000 ± 0.000 | 100 | 7815 ± 47 | 7815 ± 47 |
|  | **0.3** | 1.000 ± 0.000 | 1.000 ± 0.000 | 100 | 6069 ± 36 | 6069 ± 36 |
|  | **0.4** | 1.000 ± 0.000 | 1.000 ± 0.000 | 100 | 4601 ± 25 | 4601 ± 25 |
|  | **0.5** | 1.000 ± 0.000 | 0.998 ± 0.000 | 90 | 3326 ± 23 | 3325 ± 23 |
|  | **0.6** | 0.963 ± 0.003 | 0.997 ± 0.001 | 2 | 2200 ± 17 | 2133 ± 17 |
|  | **0.7** | 0.492 ± 0.003 | 0.231 ± 0.001 | 0 | 1394 ± 4 | 1237 ± 13 |
|  | **0.8** | 0.425 ± 0.002 | 0.057 ± 0.001 | 0 | 1301 ± 2 | 493 ± 8 |
|  | **0.9** | 0.384 ± 0.002 | 0.027 ± 0.001 | 0 | 1298 ± 2 | 51 ± 2 |
|  | **random** | 0.374 ± 0.002 | 0.020 ± 0.001 | 0 | 1291 ± 2 | 0.46 ± 0.05 |
| **1000 units, small communities** | **0.1** | 1.000 ± 0.000 | 0.995 ± 0.000 | 99 | 13433 ± 57 | 13433 ± 57 |
|  | **0.2** | 1.000 ± 0.000 | 0.988 ± 0.001 | 99 | 11071 ± 40 | 11071 ± 40 |
|  | **0.3** | 1.000 ± 0.000 | 0.981 ± 0.001 | 100 | 8795 ± 33 | 8795 ± 33 |
|  | **0.4** | 1.000 ± 0.000 | 0.971 ± 0.001 | 100 | 6708 ± 29 | 6708 ± 29 |
|  | **0.5** | 1.000 ± 0.000 | 0.958 ± 0.002 | 100 | 4982 ± 22 | 4982 ± 22 |
|  | **0.6** | 0.999 ± 0.000 | 0.933 ± 0.003 | 56 | 3451 ± 14 | 3446 ± 14 |
|  | **0.7** | 0.858 ± 0.001 | 0.732 ± 0.016 | 0 | 2114 ± 22 | 2105 ± 11 |
|  | **0.8** | 0.562 ± 0.002 | 0.123 ± 0.002 | 0 | 1334 ± 2 | 1030 ± 6 |
|  | **0.9** | 0.505 ± 0.002 | 0.058 ± 0.001 | 0 | 1293 ± 2 | 238 ± 2 |
|  | **random** | 0.486 ± 0.002 | 0.040 ± 0.001 | 0 | 1286 ± 2 | 0.36 ± 0.03 |
| **5000 units, big communities** | **0.1** | 1.000 ± 0.000 | 0.994 ± 0.000 | 100 | 81118 ± 121 | 81118 ± 121 |
|  | **0.2** | 1.000 ± 0.000 | 0.988 ± 0.000 | 100 | 67120 ± 100 | 67120 ± 100 |
|  | **0.3** | 1.000 ± 0.000 | 0.978 ± 0.000 | 100 | 54934 ± 91 | 54934 ± 91 |
|  | **0.4** | 1.000 ± 0.000 | 0.963 ± 0.001 | 100 | 43834 ± 77 | 43834 ± 77 |
|  | **0.5** | 1.000 ± 0.000 | 0.942 ± 0.001 | 100 | 33410 ± 52 | 33410 ± 52 |
|  | **0.6** | 1.000 ± 0.000 | 0.912 ± 0.001 | 85 | 24084 ± 41 | 24083 ± 41 |
|  | **0.7** | 0.991 ± 0.001 | 0.999 ± 0.000 | 2 | 15805 ± 34 | 15777 ± 33 |
|  | **0.8** | 0.586 ± 0.001 | 0.066 ± 0.003 | 0 | 8797 ± 4 | 8578 ± 20 |
|  | **0.9** | 0.536 ± 0.001 | 0.018 ± 0.000 | 0 | 8461 ± 4 | 2777 ± 8 |
|  | **random** | 0.517 ± 0.001 | 0.010 ± 0.000 | 0 | 8408 ± 4 | 0.50 ± 0.05 |
| **5000 units, small communities** | **0.1** | 1.000 ± 0.000 | 0.958 ± 0.000 | 100 | 99066 ± 112 | 99066 ± 112 |
|  | **0.2** | 1.000 ± 0.000 | 0.939 ± 0.000 | 100 | 82631 ± 94 | 82631 ± 94 |
|  | **0.3** | 1.000 ± 0.000 | 0.924 ± 0.001 | 100 | 67847 ± 91 | 67847 ± 91 |
|  | **0.4** | 1.000 ± 0.000 | 0.904 ± 0.000 | 100 | 54354 ± 77 | 54354 ± 77 |
|  | **0.5** | 1.000 ± 0.000 | 0.882 ± 0.001 | 100 | 41991 ± 49 | 41991 ± 49 |
|  | **0.6** | 1.000 ± 0.000 | 0.853 ± 0.001 | 92 | 30807 ± 40 | 30807 ± 40 |
|  | **0.7** | 0.999 ± 0.000 | 0.810 ± 0.001 | 14 | 20572 ± 27 | 20563 ± 27 |
|  | **0.8** | 0.693 ± 0.002 | 0.087 ± 0.004 | 0 | 10012 ± 66 | 11599 ± 18 |
|  | **0.9** | 0.620 ± 0.001 | 0.037 ± 0.000 | 0 | 8475 ± 4 | 4205 ± 8 |
|  | **random** | 0.600 ± 0.001 | 0.020 ± 0.000 | 0 | 8407 ± 4 | 0.51 ± 0.04 |
