# Supplementary material for: Deciphering Network Community Structure by Surprise
Source: PLoS One. 2011 Sep 1;6(9):e24195. doi: 10.1371/journal.pone.0024195 (PMC3164713; doi:10.1371/journal.pone.0024195)
Supplement: Table S2 — Details of the RC benchmark results. Same data as in Table S1, but with variations in the Degradation (D) parameter. Data for random networks of the same size are also included. (DOC) [file pone.0024195.s008.doc]

**Table S2**

| **RELAXED CAVEMAN** | **Degradation** | **NMI ± s. e. m.** | | **NMIs = 1 (%)** | ***S*_max_ ± s. e. m.** | ***S*_orig_ ± s. e. m.** |
| --- | --- | --- | --- | --- | --- | --- |
|  |  | ***S*** | **Q** |  |  |  |
|  | 10 | 1.000 ± 0.000 | 0.940 ± 0.005 | 79 | 12642 ± 146 | 12642 ± 146 |
|  | 20 | 0.999 ± 0.000 | 0.914 ± 0.007 | 43 | 8613 ± 86 | 8612 ± 86 |
|  | 30 | 0.992 ± 0.000 | 0.871 ± 0.008 | 8 | 4824 ± 39 | 4823 ± 39 |
|  | 40 | 0.972 ± 0.002 | 0.849 ± 0.008 | 0 | 3317 ± 24 | 3323 ± 24 |
|  | 50 | 0.928 ± 0.004 | 0.821 ± 0.008 | 0 | 2197 ± 12 | 2224 ± 13 |
|  | 60 | 0.864 ± 0.006 | 0.760 ± 0.008 | 0 | 1412 ± 6 | 1447 ± 8 |
|  | 70 | 0.735 ± 0.008 | 0.659 ± 0.008 | 0 | 866 ± 4 | 884 ± 4 |
|  | 80 | 0.478 ± 0.003 | 0.424 ± 0.006 | 0 | 653 ± 2 | 487 ± 3 |
|  | 90 | 0.350 ± 0.003 | 0.172 ± 0.003 | 0 | 645 ± 2 | 201 ± 1 |
| **RANDOM (ERDOS-RENYI)** | **Degradation** | **NMI ± s. e. m.** | | **NMIs = 1 (%)** | ***S*_max_ ± s. e. m.** | ***S*_orig_ ± s. e. m.** |
|  |  | ***S*** | **Q** |  |  |  |
|  | 10 | 0.327 ± 0.003 | 0.027 ± 0.001 | 0 | 552 ± 3 | 0.47 ± 0.04 |
|  | 20 | 0.327 ± 0.003 | 0.027 ± 0.001 | 0 | 556 ± 2 | 0.45 ± 0.05 |
|  | 30 | 0.332 ± 0.003 | 0.028 ± 0.001 | 0 | 564 ± 2 | 0.46 ± 0.04 |
|  | 40 | 0.323 ± 0.003 | 0.028 ± 0.001 | 0 | 564 ± 2 | 0.44 ± 0.05 |
|  | 50 | 0.327 ± 0.003 | 0.031 ± 0.001 | 0 | 575 ± 2 | 0.41 ± 0.04 |
|  | 60 | 0.328 ± 0.002 | 0.036 ± 0.001 | 0 | 592 ± 2 | 0.42 ± 0.04 |
|  | 70 | 0.334 ± 0.003 | 0.041 ± 0.001 | 0 | 613 ± 2 | 0.41 ± 0.04 |
|  | 80 | 0.331 ± 0.003 | 0.047 ± 0.001 | 0 | 621 ± 1 | 0.46 ± 0.04 |
|  | 90 | 0.215 ± 0.003 | 0.060 ± 0.001 | 0 | 642 ± 2 | 0.39 ± 0.04 |
